# Characterization of Collagen/Beta Glucan Hydrogels Crosslinked with Tannic Acid

**DOI:** 10.3390/polym13193412

**Published:** 2021-10-05

**Authors:** Marta Michalska-Sionkowska, Oliwia Warżyńska, Beata Kaczmarek-Szczepańska, Krzysztof Łukowicz, Anna Maria Osyczka, Maciej Walczak

**Affiliations:** 1 Faculty of Biological and Veterinary Sciences, Department of Environmental Microbiology and Biotechnology, Nicolaus Copernicus University in Toruń, 87-100 Toruń, Poland; oliwia.warzynska@vp.pl (O.W.); walczak@umk.pl (M.W.); 2 Faculty of Chemistry, Department of Biomaterials and Cosmetics Chemistry, Nicolaus Copernicus University in Toruń, 87-100 Toruń, Poland; beata.kaczmarek@umk.pl; 3 Institute of Zoology and Biomedical Research, Department of Biology and Cell Imaging, Faculty of Biology, Jagiellonian University, 31-007 Kraków, Poland; krzysztof.lukowicz@uj.edu.pl (K.Ł.); anna.osyczka@uj.edu.pl (A.M.O.)

**Keywords:** hydrogel, tannic acid, antimicrobial properties, collagen, β-glucan, biopolymers, HaCaT cells

## Abstract

Hydrogels based on collagen/β-glucan crosslinked with tannic acid were obtained by neutralization using dialysis. The presence of tannic acid allowed obtaining stable hydrogel materials with better mechanical properties. Tannic acid was released from matrices gradually and not rapidly. The antioxidant properties of the obtained hydrogels increased over the course of their incubation in culture media and were dependent on the concentration of tannic acid in the matrices. The obtained materials influenced dehydrogenase activity and the ATP level of pathogens. Additionally, the materials’ extracts improved the HaCaT cells’ viability. Therefore, the obtained hydrogels seem to be promising biocompatible materials which display antimicrobial properties.

## 1. Introduction

Natural polymers that are produced by living organisms serve as biodegradable matrices that contain repeating units linked together by covalent bonds. They include polypeptides, e.g., elastin, keratin, collagen, and polysaccharides, e.g., cellulose, chitin, or β-glucan [1].

Collagen (Coll) belongs to the family of fibrous proteins, constituting 30% of all proteins in mammalian organisms. There are at least 28 types of collagen with some differences in their composition and amino acid sequence. All collagens are made of triple-stranded helical molecules connected by hydrogen bonds [2]. The function of collagens includes: maintaining structural cohesion, the elasticity of connective tissue, interaction with specific receptors, and mechanical properties such as skin and ligament tensile strength [3]. Collagen is widely used as a biomaterial due to its biological properties. It is biodegradable and harmless to the biological environment. Its biodegradation products are not toxic, and they are not rejected by the human body [4]. In industry, mainly porcine and bovine collagens are used. The sources of collagens for broad applications in technology include skin, cow placenta and aorta. Fish skin, which is a waste from the food industry, can serve as an alternative source of this polymer.

Polysaccharide β-glucan (BG) contains glucose units. It is made of D-glucopyranose residues. The activity of β-glucan depends on its source, structure, and conformation. The sources of β-glucan include fungi (e.g., krestin isolated from *Coriolus versicolor*, schizophyllan isolated from *Schizophyllum commune*), grains (including barley, oat, rye), and yeasts *Saccharomyces cerevisiae* [5]. Cereal-derived β-glucans are mainly β-(1,3-) and β-(1,4) glycosidic linkages, while β-glucans from yeasts and fungi are β-(1,3-) and β-(1,6-) [6]. Different β-glucans may have different conformations: random coils, helices, rod-like shapes, worm-like shapes, and aggregates [6]. The biological activity of β-glucan is also affected by its molecular weight. Short chains of β-glucans that have low molecular weight, below 5000–10,000 Da, are usually inactive. High molecular weight β-glucans are cytotoxic, but they display antimicrobial activity, while low molecular weight β-glucans require synergistic action of interleukins to stimulate the immune response [7]. The properties of β-glucans also include anti-cancer, antibiotic-like activity, lowering blood pressure and cholesterol levels [8]. Studies have also been carried out on the use of diets containing β-glucan. 

The disadvantages of natural polymers (e.g., collagen, β-glucan) include poor mechanical and thermal properties; therefore, their modifications are necessary. One possible solution to the above is the use of a crosslinking agent-tannin. Tannins are natural phenolic compounds that can form complexes with polysaccharides and proteins. Tannins were successfully used as crosslinking agents before in modified natural polymers, e.g., gelatin, casein, and chitosan [9,10,11]. Tannins can be divided into two groups: hydrolysable tannins and condensed tannins. Hydrolysable tannins contain monosaccharides in their structure, which are esterified with gallic acid residues. Condensed tannins with a characteristic C6-C3-C6 carbon skeleton do not contain sugar in their structure [12]. Tannins have also antibacterial properties. They react with bacterial proteins causing their deactivation. Therefore, tannins are used, e.g., for intestine infections [13]. 

The aim of this work was to obtain and characterize collagen/β-glucan materials modified with tannic acid. We tested the hypothesis that the addition of tannic acid should act as a crosslinking agent and additionally provide antimicrobial properties. 

## 2. Materials and Methods

### 2.1. Collagen Isolation

Collagen was isolated from the skin of *Hypophthalmichthys nobilis—*a by-product from the food industry. Collagen was obtained according to the methodology described in our previous work [14]. Shortly, the skin was cut into small pieces and cleaned with distilled water. Non-collagenous proteins were extracted with 0.1 M NaOH for 4 days. Then, 10% butyl alcohol was used to remove the fat. The insoluble matter was extracted with 0.5 M acetic acid for 2 days. Collagen was salted out by adding NaCl (to a final concentration of 2 M). The precipitated protein was centrifuged (Rotina 38R, Hettich, Tuttlingen, Germany) at 10,000 rpm for 30 min. The resulting precipitate was dissolved in 0.5 M acetic acid and dialyzed (MWCO = 12–14 kDa, Serva, Heidelberg, Germany) against 0.1 M acetic acid for 5 days, changing the solution every 24 h. Then, the solution was frozen and lyophilized (ALPHA 1–2 LD plus, CHRIST, Osterode am Harz, Germany). Obtained collagen was used for materials preparation.

### 2.2. Hydrogel Preparation 

For hydrogel preparation, first, 1% collagen solution was prepared in 0.1 M acetic acid, and 5% of β-glucan (cosmetic grade) was prepared in distilled water and heated in a water bath without boiling. Two percent tannic acid (TA) (Sigma-Aldrich, Saint Louis, MO, USA) was prepared in distilled water. Collagen and β-glucan solutions were mixed in a 90/10 *w/w* ratio. This composition was chosen based on our previous study [14]. The collagen/β-glucan mixture was modified by using 2, 5, and 10% tannic acid addition. The hydrogels were obtained by dialysis against distilled water for 7 days, changing the distilled water every 24 h. 

### 2.3. Infrared Spectroscopy 

The interaction between functional groups of polymers and tannic acid was evaluated by attenuated total reflection infrared spectroscopy using Nicolet iS10 equipped with an ATR device with a diamond as the crystal. All the spectra were recorded in the range of 500–4000 cm^−1^, and 64 scans were obtained. The spectra were registered for Coll/BG, Coll/BG + 2%TA (after lyophilization), and tannic acid alone.

### 2.4. Mechanical Properties

**Figure 1:**
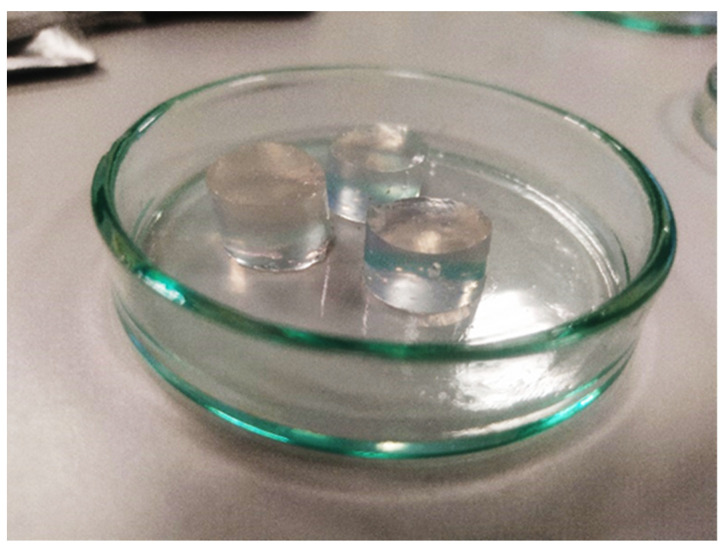
A sample photo of Coll/BG + 5%TA material, cut into a cylindrical shape for mechanical testing.

Mechanical properties were tested using the Zwick & Roel testing machine (Zwick & Roel, Ulm, Germany). The materials were cut into cylinders of 10 mm thickness and 15.8 mm in diameter (Figure 1). At least 5 samples of each kind were placed between two discs and pressed with the crosshead speed set at 0.5 mm/min. The maximum compressive strength (σ_max_) and compressive modulus (E_mod_) were evaluated. The results are presented as mean values with standard deviation.

### 2.5. Release of Tannic Acid from Hydrogels

The release of tannic acid from matrices was analyzed using the Folin-Ciocalteu method. This method is commonly used to determine the total content of phenolic compounds [15]. Hydrogels were cut into cylinders of 10 mm high and placed into separate wells in 12-well plates filled with 2 mL PBS buffer (pH = 7.4) and incubated at 37 °C for 2, 4, 6, 24, and 48 h. At each time point, 20 µL aliquot was withdrawn and mixed with distilled water up to 1600 µL total volume, and then 100 µL of Folin-Ciocalteu reagent (Sigma-Aldrich) was added. After 3 min of incubation, 300 µL of saturated Na_2_CO_3_ solution was added to the above mixture, and the final solution was kept at 40 °C until a characteristic blue color was obtained (i.e., approx. for 30 min) [16]. The absorbance was measured spectrophotometrically at 725 nm. The standard curve was prepared with gallic acid at the concentration range of 0–2.5 mg/mL (R^2^ = 0.9994). The analyses were performed for 4 replicates for each hydrogel. After each solution was withdrawn, 20 µL of fresh PBS buffer was added to keep the constant volume (2 mL) in each well. 

### 2.6. Antioxidant Activity

The antioxidant properties of the obtained hydrogels were assessed according to the method described by Ali et al. 2020, with small modifications [17]. The antioxidant properties of hydrogels were determined using the DPPH reagent (i.e., 2,2-Diphenyl-1-picrylhydrazyl, free radical, 95%; Alfa Aesar, Kandel, Germany). The DPPH assay is an electron transfer-based method that measures the capacity of an antioxidant (hydrogen donator) in the reduction of an oxidant. This method can be used to quantify antioxidants in complex biological systems for both solid and liquid samples [18]. In the presence of a free radical acceptor, the dark purple color of the solution changes to yellow, thanks to which it is possible to measure the samples with a spectrophotometer [14]. A solution of 250 µM DPPH with methanol as a solvent was prepared. The materials were cut into 5-mm high pieces, placed in separate wells in a 24-well plate and soaked in 2 mL of DPPH solution for 24 h in the dark. Three samples of hydrogel were used for this experiment. As control, the DPPH solution without any material was left in the same conditions. After 24 h of incubation, spectrophotometric measurements of the DPPH solutions were made at 517 nm. The antioxidant activity was calculated using the equation:

$$Antioxidant\;activity\;\left[\%\right]=\frac{Abs_{DPPH}-Abs_{TS}}{Abs_{DPPH}}\times 100\%$$

where:

*Abs_DPPH_* is the absorbance of the DPPH solution without contact with materials;

*Abs_TS_* is the absorbance of the DPPH solution after the contact with materials.

### 2.7. The Effects of the Materials on Bacterial Cellular Processes

Strains of *Escherichia coli* ATCC8739, *Pseudomonas aeruginosa* KKP 991, and *Staphylococcus aureus* ATCC6538 were used. Hydrogels were sterilized in a 70% ethyl alcohol–water solution for 10 min, then the alcohol was removed, and the materials were washed once with sterile PBS buffer. Finally, the hydrogels were subjected to UV light under a laminar chamber for 20 min for both sides. Sterile materials were cut using a sterile scalpel into cylinders that were 10 mm high and 15.8 mm in diameter. The bacteria were cultured in the flasks containing 20 mL of nutrient agar broth (bacteriological peptone 5 g/L; yeast extract 3 g/L; pH = 6.8–7.2) in 37 °C for 24 h. The cells were harvested by centrifugation and washed once with a sterile 0.9% NaCl solution. The supernatant was discarded, and the cells were washed again. The bacterial cells were then suspended in fresh NaCl solution to obtain optical density = 1.0 in the McFarland scale. The materials were placed in separate wells in a 24-well plate filled with 2250 µL of nutrient broth (composition: 3 g/L of yeast extract) and 250 µL of cell culture. The samples were incubated at 37 °C for 60 min. Positive control was prepared by adding the same volume of nutrient broth and cell suspension without hydrogel. After incubation, 100 µL of the medium was removed from each well and transferred to separate wells in 96-well plates for subsequent analysis. Each hydrogel sample was tested in 3 replications. The results are presented as the mean and standard deviation [19].

#### 2.7.1. The Influence of the Materials on Dehydrogenase Activity

The dehydrogenase activity was performed using the CellTiter96 AQueous reagent (Promega, Madison, WI, USA) according to the protocol provided by the manufacturer. After contact with the materials, 100 µL of bacteria cell suspension were mixed with 20 µL of the reagent and incubated at 37 °C for up to 4 h depending on the bacterial strain. After incubation, the absorbance was measured at 490 nm using microplate reader Multiscan FC (Thermo Fisher Scientific, Waltham, MA, USA). The results are presented as percent values using control as 100%.

#### 2.7.2. The Influence of Materials on ATP Level

Adenosine-5′-triphosphate (ATP) levels were analyzed using BacTiter-Glo^TM^ (Promega, Madison, WI, USA). All the steps were performed in accordance with the protocol provided by the manufacturer. Shortly after contact with materials, 100 µL of bacteria cell suspension were mixed with the same volume of a reagent. The samples were then incubated on an orbital shaker for 5 min. The luminescence of the samples was measured using Synergy HT Multi-Mode Microplate Reader (BioTek Instruments, Winooski, VT, USA). The results are presented as percent values using control as 100%.

### 2.8. Effects of the Material Extracts on HaCaT Cultures

For biocompatibility studies, the materials were sterilized as described in Section 2.7. The HaCaT human keratinocyte line (Thermo Fisher Scientific, Waltham, MA, USA) was selected for the initial evaluation of the materials. Unless stated otherwise, the cell culture reagents were purchased from Thermo Fisher Scientific. The material extracts were prepared by 72 h of incubation of the materials in a growth medium (1 mL/well/material) composed of alpha-minimum essential medium (α-MEM) supplemented with 10% fetal bovine serum (FBS) and antibiotics (penicillin/streptomycin 1% solution). The cells were seeded on tissue culture plastic (TCP) at the density of 2 × 10^4^ cells/cm^2^ and cultured for 24 h. Then, the medium was removed, the cells were washed with PBS and exposed to the 1 ml extracts/well for an additional 24 h and then assessed for viability. The cells cultured on TCP without any stimulation were considered as a general reference. To assess the cells’ viability, the cultures were washed with PBS and 0.2 mL solution of 10% MTS reagent (CellTiter96Aqueous One Solution Cell Proliferation Assay; Promega) in phenol-free alpha-MEM was added to individual wells. The plates were incubated at 37 °C until an apparent change of color from yellow to brownish. Then, the media were transferred to individual wells in 96-well plates, and the absorbance was recorded at 492 nm using a plate reader. The materials were analyzed in three repetitions.

### 2.9. Statistical Analysis

The data were analyzed by one-way ANOVA using Tukey post hoc tests (*p* ≤ 0.05) (Graph Pad Prism 7.05 software, La Jolla, CA, USA), and *p* < 0.05 was considered significant. 

## 3. Results

### 3.1. Infrared Spectroscopy 

**Figure 2:**
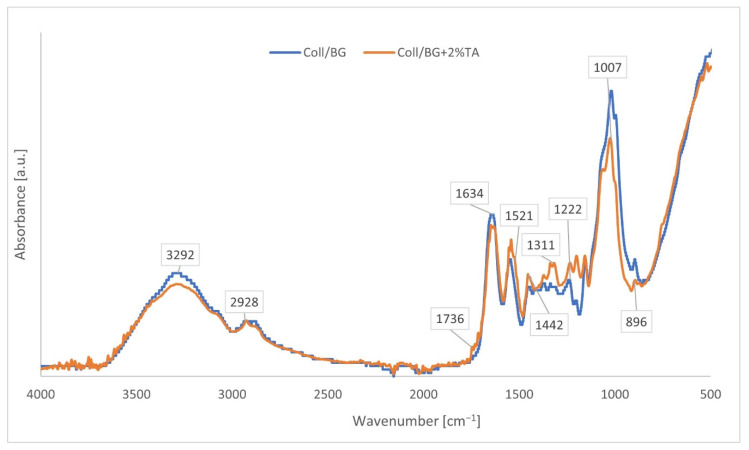
ATR-IR spectra for Coll/BG with 2 wt.% of tannic acid (Coll/BG + 2%TA) and without it.

ATR-FTIR spectroscopy was applied for the identification of the chemical structure of the compounds. The ATR-FTIR spectra of Coll/BG and Coll/BG + 2%TA are shown in Figure 2. The Coll/BG spectrum showed groups such as Amide A (3292 cm^−1^), Amide B (2928 cm^−1^), Amide I (1634 cm^−1^), Amide II (1521 cm^−1^), Amide III (1222 cm^−1^) characteristic for collagen samples. The maximum peak at 1007 cm^−1^ corresponded to glucopyranose moiety, and at 896 cm^−1^, a characteristic of β-linked glycosidic bonds constituted characteristic adsorption peaks for β-glucan. Those peaks were also present in the spectra crosslinked with tannic acid. 

Additionally, a small peak at 1736 cm^−1^ was observed that indicated the presence of a carboxyl carbonyl group. The peak at 1442 cm^−1^ implied the deformation of –C–C– in the phenolic groups, while the band at 1311 cm^−1^ was due to the presence of an aromatic group –C–O. ATR-FTIR spectra of Coll/BG crosslinked with tannic acid showed a shift towards higher values from 1521 to 1539 cm^−1^ that corresponded to NH_2_ bond stretching (Amide II). 

### 3.2. Mechanical Properties

**Figure 3:**
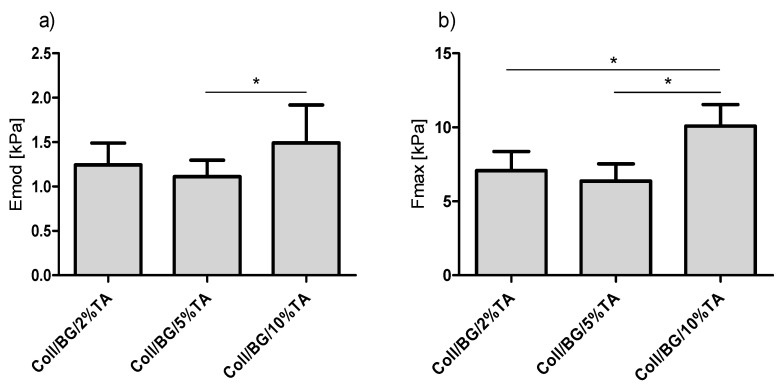
The mechanical parameters of hydrogels with different tannic acid concentrations: (**a**) comprehensive modulus; (**b**) maximum tension; * *p* < 0.05 between groups.

The results of mechanical parameters tests are presented in Figure 3. Although some increase in the comprehensive modulus (E_mod,_ Figure 3a) with the increase in tannic acid content could be noticed in some samples, these differences were not statistically significant (*p* <0.05). The maximum tension (σmax, Figure 3b) increased after the addition of 10% of tannic acid, but this was not statistically significant (*p* > 0.05). Additionally, no statistically significant changes in the maximum tension values were observed for hydrogels with 2% and 5% TA. 

### 3.3. Tannic Acid Release 

**Figure 4:**
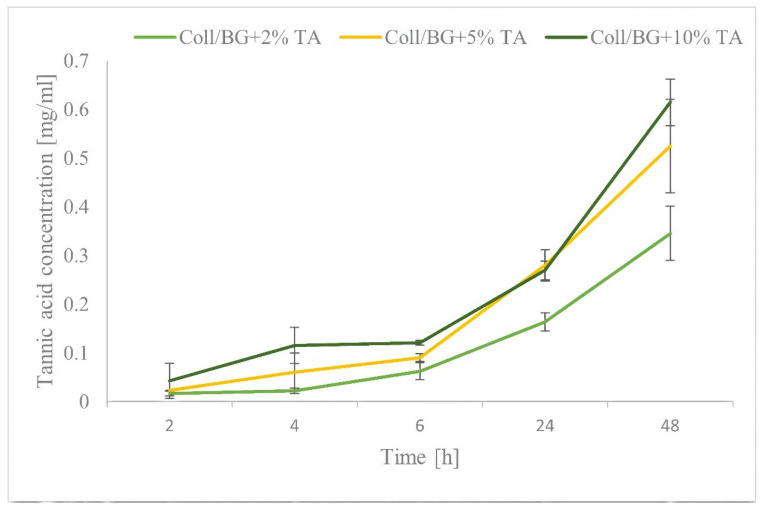
The tannic acid release rate from hydrogels.

The release rate of the tannic acid is shown in Figure 4. The concentration of the released tannic acid depended on the initial content of tannic acid in the hydrogel. The released amount of TA increased with the time of the samples’ immersion in PBS. We observed a higher release of TA with higher concentrations of tannic acid in the hydrogel. The tannic acid was released gradually over time; there were no sudden bursts in the release of polyphenol from the matrix. The latter is an advantage because it allows for the construction of controlled delivery systems. 

### 3.4. Antioxidant Activity

Table 1 shows the antioxidant properties of the prepared materials measured as a percentage of free radical scavenging. The antioxidant properties depended on the tannic acid concentration and the sample’s incubation time. After 1.5 h of incubation, the antioxidant activity of the hydrogels containing 2% or 5% tannic acid was approx. 1%, whereas for hydrogels containing 10% tannic acid free radical scavenging was 7.91%. This corresponds to the gradual release of tannic acid from the matrices. After 18 h of contact with the material, the antioxidant activity increased significantly compared to 1.5 h incubation time for the materials containing 5% or 10% of tannic acid, which were 62.08% and 74.83%, respectively. For the material containing 2% of tannic acid, the antioxidant activity was still less than 1% after 18 h of incubation; however, after 24 h incubation, it increased to 20.16%. The material Coll/BG + 10% TA after 24 h incubation time had the highest values of free radicals scavenging (81.06%).

### 3.5. The Influence of the Materials on the Dehydrogenase Activity of Bacteria Cells

**Figure 5:**
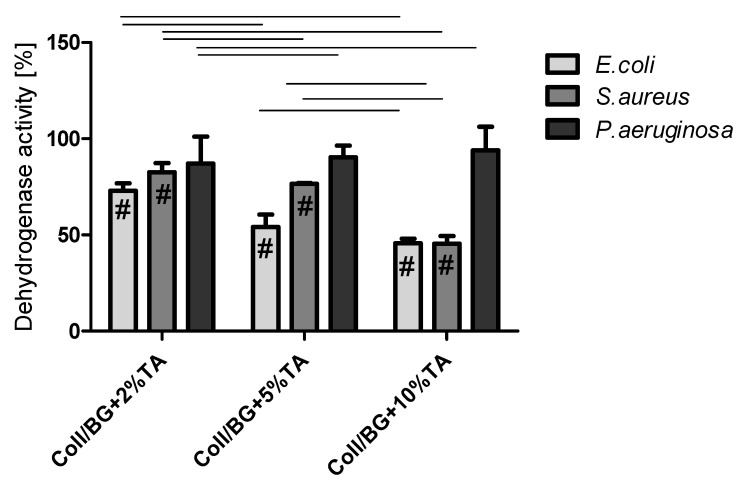
Dehydrogenase activity of *Escherichia coli*, *Staphylococcus aureus*, and *Pseudomonas aeruginosa* after 1 h of contact with hydrogels (#—significant difference from the control; lines between bars mark significant differences between groups.

Dehydrogenases are enzymes belonging to the oxidoreductases class. They catalyze the oxidation-reduction reaction with the participation of the NAD+/NADP+ coenzyme or flavins such as FAD, FMN as an electron acceptor [20]. The dehydrogenase activity level of the studied bacteria strains after contact with hydrogels is shown in Figure 5. The results are presented as the percentage of dehydrogenase activity relative to the positive control expressed as 100%. The dehydrogenase activity was inhibited at the range of 6–54%, depending on the bacterial strain. Increased content of tannic acid in the studied materials correlated with decreased activity of the dehydrogenases of *E. coli* and *S. aureus*. Coll/BG + 10%TA inhibited the dehydrogenase activity by approx. 55% after 60 min of the contact of the bacterial suspension with the materials. *E. coli* were the most sensitive to the action of the materials. The results obtained by Kaczmarek et al. 2020 also showed that materials containing tannic acid inhibited the dehydrogenase activity of *E. coli* and *S. aureus* [15]. In their studies, the increased concentration of tannic acid in the studied materials resulted in higher inhibition of the dehydrogenase activity of the tested bacteria strains. Furthermore, *E. coli* and *S. aureus* dehydrogenase activity was also examined after 2 h of contact with magnetic nanoparticles covered with lysozyme/tannic acid (Lys/TA)_5_ and lysozyme/tannic acid and silver nanoparticles (Lys/TA)_5_-Ag [21]. These authors observed high dehydrogenase activity for the control sample, and the dehydrogenase activity decreased after (Lys/TA)_5,_ whereas no enzyme activity was observed after the contact with (Lys/TA)_5_-Ag for both *E. coli* and *S. aureus* [21]. Our results were similar to the results presented in the literature. Tannic acid addition into materials influenced the dehydrogenase activity of pathogens. 

### 3.6. The Influence of the Materials on ATP Level in Selected Bacteria Strains

**Figure 6:**
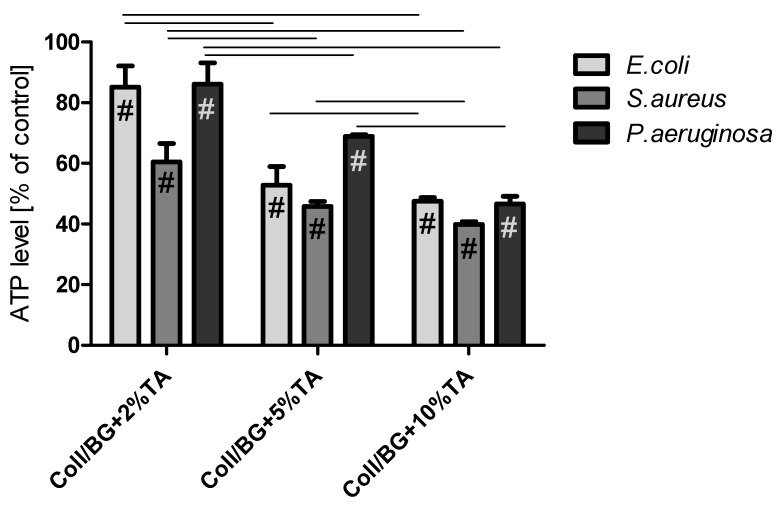
Released ATP levels of *Escherichia coli*, *Staphylococcus aureus*, and *Pseudomonas aeruginosa* after 1 h contact with hydrogels (#—significant difference with the control; lines between bars mark significant differences between groups).

Adenosine triphosphate (ATP) is produced only by metabolically active cells [22]. The bacterial ATP levels after 1 h of contact with the hydrogels are shown in Figure 6. The ATP levels decreased with increased TA concentration in the hydrogels. Coll/BG + 2%TA decreased the bacterial ATP level by approx. 15% for *E. coli* and *P. aeruginosa* and by approx. 40% for *S. aureus*. Hydrogels containing 5% TA decreased the ATP level in the range of 32–48%, depending on the strain. The 10% TA addition to the hydrogels was the most effective and caused a decrease of approx. 50% in the ATP level in all the tested strains. 

### 3.7. Biocompatibility Studies in HaCaT Cultures

**Figure 7:**
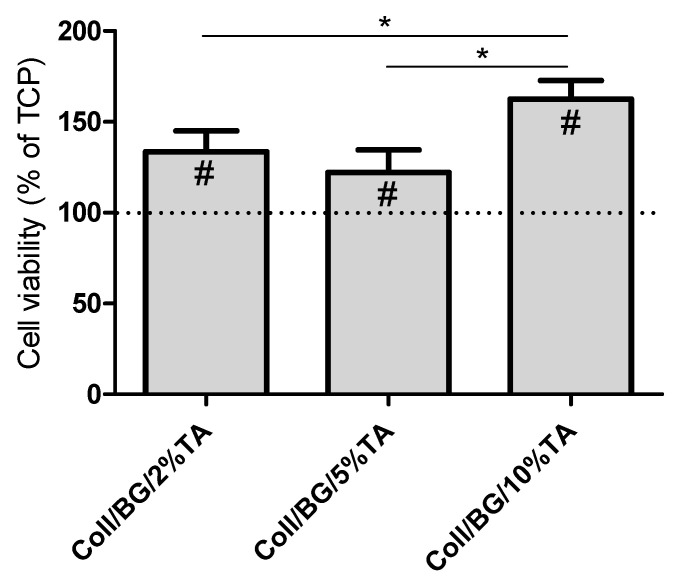
Viability of HaCaT cells after 24-h exposure to the materials’ extracts prepared by 72-h soaking of the hydrogels in a culture medium. (* *p* < 0.05 between groups; #—significant difference with the control).

The human keratinocytes’ (HaCaT cell line) viability is presented in Figure 7. Previously, we showed that materials based on the fish skin collagen and β-glucan were not toxic when composed at the 90/10 ratio of Coll/BG [15]. In this work, all the extracts obtained from the studied hydrogels increased the HaCaT cells’ viability compared to TCP, namely by 20–60% compared to TCP, depending on the TA content. The highest cell viability could be observed for Coll/BG + 10%TA, and it was significantly higher than for the respective hydrogels with 2 and 5% of TA.

## 4. Discussion 

In this study, the hydrogels based on collagen and β-glucan modified with tannic acid were obtained using dialysis as a neutralization process. Tannic acid was used as a crosslinking agent, which additionally had a positive effect on the microbiological and biological properties of the prepared materials. Therefore, using tannic acid as a crosslinking agent is a good strategy to obtain multifunctional dressings.

ATR-FTIR spectroscopy may be used to analyse the interaction between functional groups of the polymers used. Tannic acid may interact with polysaccharides (e.g., chitosan); these interactions were attributed to hydrogen bonds and van der Waals interactions between O-H groups of tannic acid and N-H groups of chitosan [23]. The molecular interaction between tannic acid and proteins (e.g., gelatine) was also shown by observing the shifts attributed to N-H and O-H partaking in hydrogen bonds [24]. We did not observe any significant shifts in the FTIR spectra (Figure 2); however, based on the literature we assume that between the hydrogel components, hydrogen bonds and van der Waals interactions are present.

Materials based on natural polymers have several advantages, such as biocompatibility, biodegradability, and non-toxicity; however, they mostly show poor mechanical properties [25]. One way to improve the latter is to conduct a physical, chemical, or enzymatic crosslinking process. Improvement of the mechanical properties of materials after a crosslinking process is often observed [26,27,28]. Crosslinking provides a more complex structure of the polymer chain and additional bonds. In this study, we did not observe any significant changes in the comprehensive modulus, and only the maximum tension increased after using 10% tannic acid concentration to crosslink chitosan hydrogels (Figure 3). Despite the use of a crosslinking agent (glyoxal), no statistically significant differences were observed in the comprehensive modulus, the maximum tension, and the percentage of deformation at the maximum tension of chitosan hydrogels after modification with different concentrations of tannic acid [29]. Additionally, hydrogel gelatin nanoparticles crosslinked at different times with glutaraldehyde had no influence on the Young’s modulus values [27]. However, Ahammed et al. (2021) observed improved mechanical properties of gelatin/zein films crosslinked with transglutaminase that were proportional to the crosslinking ratio [30]. Thus, the crosslinking process may influence the mechanical properties of some materials. The crosslinking agent concentration in this study had no effect on the mechanical properties of Coll/BG. However, considering that the hydrogels made only with Coll/BG were not solid and thus it was impossible to use them for comparison, we can assume that crosslinking improved the mechanical properties of all the prepared crosslinked hydrogels. 

Bacterial contamination of a wound might occur at any time during the wound healing process; therefore, prolonged antimicrobial agent release from the wound dressing is beneficial [31]. We believe that prolonged release of tannic acid may be beneficial to the antibacterial properties of a material. No sudden release of tannic acid was observed by Kaczmarek et al. (2020) with the materials based on sodium alginate and tannic acid, where the release of tannic acid was gradual and depended on the concentration of this polyphenol in the matrix [15]. The opposite situation was observed by Kuai et al. (2020), where the release of phenols to food simulants were rapid during early incubation stages and slow at the later incubation stages [32]. Rapid release of an active agent from hydrogel materials was noticed by Fan et al. 2021 [33]. Conversely, Séon-Lutz et al. (2019) observed that the profile of drug release from matrices depended on the thickness of the materials [34]. They observed that, for films with 0.15 mm thickness, naproxen did not penetrate deep into the matrices, but most probably, it was located near the surface, which allowed for its fast release. The larger surface-to-volume ratio allowed the penetration of the drug into the core of the fibers, and a sustained release of naproxen in higher quantities was observed [34]. In our study, gradually releasing tannic acid over time (Figure 4) is an advantage because it allows for the construction of controlled delivery systems. 

It was shown that the excess of reactive oxygen species (ROS) during the wound repair process often impairs wound healing by changing or degrading extracellular matrix (ECM) proteins, damaging dermal fibroblasts, and reducing the function of keratinocytes [35]. Thus, by controlling the ROS level, wound healing may be rendered more effective [36]. It was observed that the antioxidant properties of some medical plants depend on the total phenolic content. Probably, the antioxidant properties depend on their redox properties, and this can play an important role in neutralizing free radicals, quenching singlet and triplet oxygen, or decomposing peroxides [37]. Fan et al. (2017) observed that adding tannic acid to hydrogel materials resulted in antioxidant and immunomodulatory properties of the materials, thanks to the presence of free radical scavengers [38]. The ability of tannic acid to scavenge free radicals is based on the presence of phenolic hydroxyl groups that are able to reduce DPPH free radicals. As the concentration of tannic acid in the material increases, it is possible to obtain materials presenting high antioxidant activity—providing that the TA is released to the environment [39]. Our results showed that the studied hydrogels containing tannic acid display strong antioxidant properties based on free radical scavenging (Table 1). 

In 1987, Gristina formulated the phrase “race for the surface” to describe the competition for surface colonization between somatic and bacteria cells [40]. Bacterial cells have an advantage in this race due to their ability to colonize both biotic and abiotic surfaces [41]. An open wound is a niche that is favorable for microbial colonization. Moreover, because of the increasing antibiotic resistance and infections, longer hospital stays and expensive intensive care is needed [42]. Therefore, plenty of efforts have been made to develop antimicrobial wound dressing. In this study, the addition of tannic acid decreased the dehydrogenase activity of *E. coli* and *S. aureus* and decreased the ATP level in *E. coli*, *S. aureus* and *P. aeruginosa* (Figure 5 and Figure 6). This depended on the tannic acid concentration in the matrix. Dehydrogenase belongs to the enzymes participating in the electron transport chain, during which energy in the form of ATP is generated. Therefore, the dehydrogenase activity and ATP levels are connected. In our previous work, we observed the correlation between dehydrogenase activity and ATP level in *S. aureus*, *E. coli*, and *P. aeruginosa* bacteria strains after their treatment with materials containing thymol [19]. In this work, regarding Coll/BG + TA hydrogels, this situation could be observed for *E. coli* and *S. aureus* strains but not for *P. aeruginosa*. For the latter, the dehydrogenase activity was very high for each studied sample, and ATP levels decreased with increasing tannic acid addition. We suspect that tannic acid did not affect dehydrogenase activity but increased the hydrolysis of ATP. During the electron transport chain, ATP is produced. Therefore, the lower ATP level may be a result of the reduced rate of ATP synthesis. Moreover, a lower ATP level may be caused by a higher exhaust of intracellular ATP used by cells in an attempt to regenerate the proton motive force [43]. Thus, *Pseudomonas aeruginosa* probably used higher amounts of ATP to defend from damage and to carry out cell repair. The lower ATP level may be caused by s higher exhaust of intracellular ATP used by cells in an attempt to regenerate the proton motive force [43]. 

Considering materials as wound dressing, the important factor is their biocompatibility. In our study, we showed that materials with tannic acid increase the HaCaT cells’ viability compared to TCP (Figure 7). Our results were similar to the results presented by other authors. Tannic acid hydrogels crosslinked by trimethylolpropane triglycidyl ether obtained by Sahiner et al. (2016) were characterized by antimicrobial properties, and, at the same time, they were biocompatible in the cultures of L929 fibroblast cells [44]. It is crucial to find a balance between antimicrobial properties and biocompatibility. Materials should be antimicrobial, but, at the same time, they cannot be toxic for human cells. Zhang et al. (2020) obtained bacterial cellulose/tannic acid composites with MgCl_2_ additives. They confirmed antimicrobial and antibiofilm properties of such materials against *P. aeruginosa* and *S. aureus* and showed their biocompatibility in cultures of L929 cells [45]. Probably, the controlled release of tannic acid from composites provides less cytotoxic effects for body cells. Therefore, using tannic acid as a component of materials may prove effective not only as a crosslinking agent but also as an antimicrobial agent that does not show cytotoxicity to human cells. 

## 5. Conclusions

Tannic acid was successfully used as a crosslinking agent for collagen/β-glucan hydrogels. The presence of tannic acid allowed obtaining stable hydrogel materials with better mechanical properties. The release of tannic acid was prolonged, which may act beneficially for the biological properties of hydrogels. Moreover, the use of tannic acid resulted in obtaining materials presenting some antimicrobial properties. The obtained materials influenced dehydrogenase activity and the ATP level of pathogens. as Additionally, they showed good antioxidant properties. Moreover, hydrogels resulting in improved HaCaT cells’ viability. Therefore, we believe the obtained hydrogels may prove promising materials that are antimicrobial and biocompatible at the same time. 

## Figures and Tables

**Table 1:** The antioxidant properties of the hydrogels during different incubation times.

Specimen	After 1.5 h of Contact [%]	After 18 h of Contact [%]	After 24 h of Contact [%]
Coll/BG + 2%TA	0.23 ± 0.01	0.84 ± 0.04	20.16 ± 0.08
Coll/BG + 5%TA	0.91 ± 0.03	62.08 ± 0.12	58.30 ± 0.17
Coll/BG + 10%TA	7.91 ± 0.02	74.83 ± 0.24	81.06 ± 0.28

## Data Availability

The data presented in this study are available on request from the corresponding author.
